# Work-related psychosocial risk factors for intensive care unit nurses and practical nurses in a general hospital

**DOI:** 10.47626/1679-4435-2025-1431

**Published:** 2025-08-25

**Authors:** Daniele Almeida Duarte, Ellen Ferrari Silveira, Jéssica Syrio Callefi

**Affiliations:** 1 Departamento de Psicologia, Universidade Estadual de Maringá, Maringá, PR, Brazil; 2 Psicologia Hospitalar, Hospital Universitário Júlio Muller, Cuiabá, MT, Brazil; 3 Engenharia de Produção, Universidade de São Paulo, São Carlos, SP, Brazil

**Keywords:** psychosocial impact, mental health, working conditions, nurses, licensed practical nurses, impacto psicossocial, saúde mental, condições de trabalho, enfermeiras e enfermeiros, técnicos de enfermagem

## Abstract

**Introduction:**

The restructuring of the capitalist mode of production under neoliberalism has led to new forms of work organization, transforming the health-disease process of individuals, particularly work-related mental health.

**Objectives:**

To identify the psychosocial factors at work affecting nurses and practical nurses across intensive care units in a general hospital located in the Midwest region of Brazil.

**Methods:**

This is a descriptive, cross-sectional study with a quantitative approach. Data collection instruments were administered via Google Forms from August to September 2023.

**Results:**

Our analysis highlighted medium psychosocial risks arising from the Division of Tasks, Lack of Recognition, and Psychological, Social, and Physical Harms factors. The factors that require immediate attention are Social Division of Labor and Mental Exhaustion. There is a clear need for interventions focused on enhancing communication and relationships between team members and managers, preventing prejudice, discrimination, and abuse in the workplace, improving work organization and overall working conditions, and prioritizing the enhancement of free/leisure time, sleep, and food intake.

**Conclusions:**

It is essential to develop initiatives focused on health care, prevention, and health promotion by identifying and analyzing potential risks and harms affecting the health of health care workers. The findings of this study aim to support the development, enhancement, and reinforcement of policies and programs aimed at improving the well-being of health professionals.

## Abstract (pt)

**Introdução:**

A reestruturação do modo de produção capitalista neoliberal resultou em novas formas de organização do trabalho, transformando o processo saúde-doença dos indivíduos, especialmente no que se refere à saúde mental relacionada ao trabalho.

**Objetivos:**

Identificar os fatores de risco psicossociais no trabalho presentes no contexto de enfermeiros e técnicos de enfermagem atuantes em unidade de tratamento intensivo de um hospital geral da região Centro-Oeste do Brasil.

**Métodos:**

Estudo descritivo, transversal, de abordagem quantitativa. A coleta de dados foi realizada por meio da plataforma Google Forms, entre agosto e setembro de 2023.

**Resultados:**

Destacaram-se como focos de atenção os riscos psicossociais médios relacionados à divisão de tarefas, à falta de reconhecimento e aos danos psicológicos, sociais e físicos. Os fatores que demandam intervenções imediatas são a divisão social do trabalho e o esgotamento mental. Evidencia-se a necessidade de ações voltadas ao aperfeiçoamento da comunicação e das relações entre equipe e gestão; à prevenção de preconceitos, discriminações e violências no contexto laboral; ao aprimoramento da organização e das condições de trabalho; e à melhoria, principalmente, do tempo livre/lazer, sono e alimentação.

**Conclusões:**

É indispensável o desenvolvimento de iniciativas de cuidado, prevenção e promoção da saúde, a partir da identificação e análise dos possíveis riscos e agravos à saúde desses trabalhadores. Os achados desta pesquisa visam subsidiar a construção, ampliação e fortalecimento das políticas e programas voltados à saúde do trabalhador de saúde.

## INTRODUCTION

Despite the extensive knowledge gained from research, scientific evidence, and the efforts of occupational health professionals, the relationship between work and health remains dominated by the logic of capital. This focus often conflicts with the well-being of the workforce, prioritizing profit over the integrity, protection, health, and safety of workers. The dismantling of social protections, such as labor, social security, and welfare rights, exacerbates this scenario. It degrades citizenship and human rights through flexible labor relations, weakens protective institutions, and individualizes risks, leaving workers socially vulnerable.^^1^,^2^^

The restructuring of the capitalist mode of production under neoliberalism has led to new forms of work organization, transforming the health-disease process of individuals. Problems related to psychosocial factors at work (PFWs) have become emblematic in this context, causing numerous health issues for workers and posing a challenge for prevention, protection, and intervention efforts both in the workplace and within health care networks.^^2^,^3^^ In this scenario, mental and behavioral disorders resulting from exposure to PFWs are now a major component of the Brazilian Ministry of Health’s list of work-related diseases (updated in 2020 and republished in 2023). Additionally, these disorders are among the leading causes of work incapacity benefits in Brazil, despite persistent underreporting and difficulties in establishing their direct link to work.^^3^^

Understanding and addressing the work process of PFWs poses a significant challenge. The International Labor Organization (ILO) defines PFWs as the interaction between aspects of work-such as planning, organization, management, work environment, job content, and organizational conditions-and workers, including their capacities, needs, expectations, customs, and personal extra-job conditions. This dynamic process affects workers’ health, well-being, performance, and job satisfaction.^^4^^

The term is polysemic, evolving, and lacks a uniform definition. Therefore, to fully understand PFWs, it is essential to examine the organization and content of the work process, where the variables that determine psychosocial risks emerge. This involves considering the work environment, conditions, and relationships, all of which can impact well-being and potentially lead to pain, injuries, accidents, illnesses, and suffering for workers.^^2^,^5^^

PFWs are uniquely complex in the health sector, especially given the immaterial dimension of health care. This field involves relationship aspects, incorporating bodily, intellectual, creative, affective, and communicational practices intricately linked to individuals’ subjective experiences. Furthermore, health care work has a collective dimension, comprising multiple professional categories, qualifications, functions, positions, skills, abilities, and specializations. Educational levels and employment relationships are also diverse.^^2^,^6^^

In Brazil, nursing is regulated by Law 7,498 of 1986, which assigns nurses the management of category bodies. Their responsibilities include leadership, organization, and direction of nursing services and units; planning, organization, coordination, execution, and evaluation of nursing care services; and care of greater technical complexity, which requires scientific knowledge and the ability to make immediate decisions; among others. Practical nurses, with a high school-equivalent education, participate in nursing care programs, perform nursing care actions (except those exclusive to nurses), guide and supervise nursing work at an auxiliary level, and are integral members of health care teams.^^7^^

Nursing practices concentrate on care across multiple dimensions of health. Despite this focus, the organization and division of labor often lead to fragmented and hierarchical practices, which create inequalities in the perceived social value of different roles, leading to tensions and conflicts among workers.^^8^^

Among the various professional settings, hospitals pose a particularly high risk of burnout and illness for nursing staff. This risk arises form factors such as heavy workload, low pay, shift-based work schedules including on-call shifts (leading to multiple job-holding and long working hours), unstable contracts, continuous exposure to multiple workplace hazards, vertical power structures that can encourage moral harassment, limited autonomy, individualization of work, and a lack of collective support.^^9^-^12^^

Nursing professionals fulfill diverse roles across various sectors within hospitals, notably in the intensive care unit (ICU). According to Nascimento,^^13^^ since their establishment within health facilities in the 1970s, ICUs have been dedicated to delivering mediumto high-complexity care to critically ill patients, supported by specialized multidisciplinary teams. These units are private, complex environments equipped with advanced technology for continuous patient monitoring, support, and treatment. Nursing professionals in the ICU must remain vigilant and responsive, due to the potential for emergencies. The demanding and inflexible routines of this environment require them to provide care with speed and precision.^^10^^

In view of the foregoing, the objective of this study was to identify the PFWs affecting nurses and practical nurses across 4 ICUs in a general hospital located in the Midwest region of Brazil. Currently, proactively identifying and addressing occupational hazards is essential for preventing work-related diseases and promoting health in the workplace, particularly mental health, thus enabling the implementation of effective interventions.

## METHODS

### STUDY DESIGN

This is a descriptive, cross-sectional study with a quantitative approach.^^14^^ Data collection instruments, which included sociodemographic, occupational, and personal data information, alongside the Protocol for Psychosocial Risk Assessment at Work (Protocolo de Avaliação de Riscos Psicossociais do Trabalho, PROART)^^15^^ were administered via Google Forms from August to September 2023.

Reliability analysis was performed using SPSS, version 22.0 for Windows, and showed an adequate Cronbach’s alpha of 0.881.^^16^^ For a population of 97 workers, a sample of 35 participants provided a margin of error greater than 11.14% with a 90% confidence level. The response rate for this study was 36%.

### PARTICIPANTS

The study included 35 nurses and practical nurses from our general hospital who worked in the general ICU, coronary ICU, neonatal ICU, and private/health insurance ICU during the data collection period. All participants were exclusively assigned to the ICUs. Demographic data are presented in Table 1, while occupational data are shown in Table 2. Participants’ personal data are presented in Table 3.

**Table 1:** Demographic data of the study population (n=35)

Variable	Frequency (n)	Percentage (%)
Age (years)		
18 to 24	3	8.6
25 to 34	12	34.3
35 to 44	8	22.9
45 to 54	11	31.4
55 to 64	1	2.9
Sex		
Female	27	77.1
Male	8	22.9
Sexual orientation		
Heterosexual	26	74.3
Homosexual	5	14.3
Prefer not to say	4	11.4
Marital status		
Married/consensual union	16	45.7
Separated/divorced	3	8.6
Single	15	42.9
Widowed	1	2.9
Do you have children?		
Yes	26	74.3
No	9	25.7
Color/race		
White	8	22.9
Brown (mixed race)	21	60.0
Black	6	14.7
Level of education		
Complete high school	19	54.3
Bachelor’s degree	8	22.9
Complete high school or technical degree	1	2.9
Master’s degree or higher	7	20,0
Monthly income (no. of minimum monthly salaries)^*^		
Up to 1	4	11.4
1 to 3	24	68.6
3 to 5	5	14.3
More than 5	2	5.7

* Brazilian minimum monthly salary = BRL 1,320.00 - Base year 2023.

**Table 2:** Occupational data (n=35)

Variable	Frequency (n)	Percentage (%)
Professional category		
Nurse	10	28.6
Practical nurse	25	71.4
Type of ICU		
Private/health insurance	7	20.0
Coronary	9	25.7
General	7	20.0
Neonatal	12	34.3
Shift		
Daytime (7am to 7pm)	25	71.4
Nighttime (7pm to 7am)	10	28.6
Length of service at the current hospital (years)		
Up to 1	6	17.1
1 to 3	6	17.1
3 to 5	12	34.3
5 to 10	7	20.0
More than 10	4	11.4
Do you have another employment relationship?		
Yes	25	71.4
No	10	28.6
Total working hours (weekly hours)^*^		5.7
21 to 30	2	5.7
31 to 40	9	25.7
41 to 50	14	40.0
51 to 60	3	8.6
More than 61	7	20.0
Breaks during the working day		
1 to 2 times	26	74.3
3 to 4 times	2	5.7
No breaks	7	20.0

ICU = intensive care unit.

* Considering all employment relationships.

**Table 3:** Participants’ personal data (n=35)

Variable	Frequency (n)	Percentage (%)
Do you feel respected by your immediate superior?		
No	6	17.1
Yes	29	82.9
Have you ever experienced discrimination in the workplace?		
No	27	77.1
Yes	8	22.9
Have you ever experienced some form of abuse in the workplace?		
No	22	62.9
Yes	13	37.1
Are you or have you been undergoing psychological/psychiatric treatment?		
No	27	77.1
Yes	8	22.9
Do you use any regular medications?		
No	26	74.3
Yes	9	25.7
Do you have a history of occupational accidents?		
No	27	77.1
Yes	8	22.9
Do you exercise regularly?		
No	26	74.3
Yes	9	25.7
How do you rate your free/leisure time?		
Excellent	3	8.6
Good	11	31.4
Fair	11	31.4
Poor	6	17.1
Very poor	4	11.4
How do you rate the quality of your sleep?		
Excellent	2	5.7
Good	8	22.9
Fair	12	34.3
Poor	10	28.6
Very poor	3	8.6
How do you rate the quality of your food intake?		
Excellent	0	0.0
Good	13	37.1
Fair	15	42.9
Poor	4	11.4
Very poor	3	8.6

### INSTRUMENTS AND VARIABLES

PROART was the primary tool used for our analysis.^^15^^ Interpretation of the results is based on the following criteria: (a) Work Organization, Indicators of Distress at Work, and Work-Related Harms scales - scores between 3.70 and 5.00 indicate high psychosocial risk, requiring immediate intervention, scores between 2.30 and 3.69 represent medium risk, and scores between 1.00 and 2.29 indicate low risk, which is considered a positive outcome; (b) Management Style scale - a mean score of 3.00 indicates a moderate presence of a particular style, values above 3.50 suggest a predominance of that style, and values below 2.50 indicate limited representation of the style.

The Work Organization scale assesses the Division of Tasks, considering pace, deadlines, and work execution conditions, and the Social Division of Labor, which focuses on standards, communication, evaluation, autonomy, and participation. The Management Style scale analyzes the Individualistic Style, characterized by centralized decision-making, strong bureaucracy, disciplinary rigidity, and low professional recognition, in contrast to the Collectivist Style, which values interpersonal relationships, creativity, innovation, hierarchical flexibility, and worker recognition.^^15^^

The Indicators of Distress at Work scale assesses factors such as Meaninglessness at Work, characterized by feelings of uselessness; Mental Exhaustion, related to injustice, discouragement, and exhaustion; and Lack of Recognition, associated with devaluation and limited freedom to express opinions. Lastly, the Work-Related Harms scale investigates Psychological Harms, which manifest as negative feelings about oneself and life; Social Harms, linked to isolation and difficulties in family and social relationships; and Physical Harms, evidenced by body pain and biological disorders.^^15^^

### ETHICAL CONSIDERATIONS

This study was reviewed and approved by the local Ethics Committee (approval number 6.202.557) and followed the regulatory standards for research involving human participants, as outlined in Resolutions No. 466/2012 and No. 510/2016 of the Brazilian National Health Council. Prior to inclusion in the study, all participants were informed of the details of the study via a provided link and subsequently gave their written informed consent.

## RESULTS

**Figure 1:**
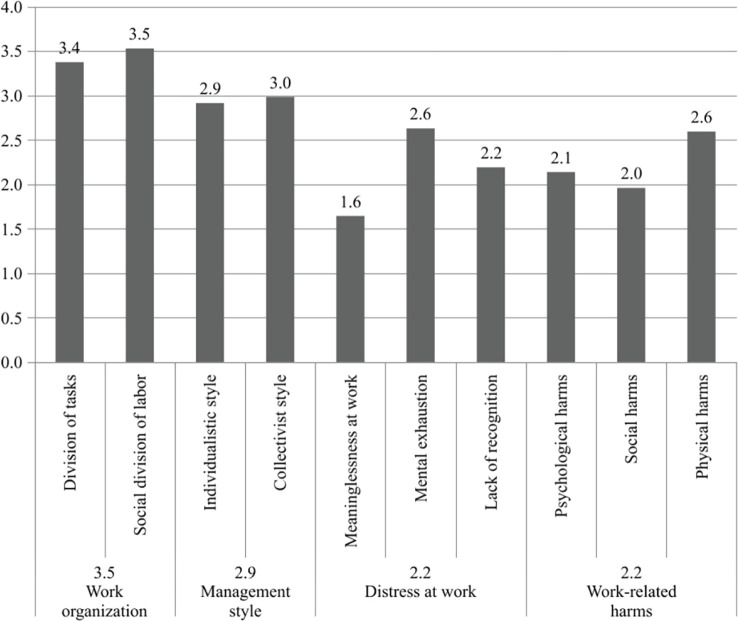
Results of the Protocol for Psychosocial Risk Assessment at Work (PROART).

The mean scores obtained in each of the PROART analysis factors are shown in Figure 1.

Based on the combination of factors assessed by the PROART scales and the variables from the sociodemographic, occupational, and personal data questionnaire, our results indicate that workers generally face a medium psychosocial risk concerning the Division of Tasks. We found evidence of high risk for those aged 18 to 24 years, separated or divorced individuals, and those with either up to 1 year or more than 10 years of employment at the institution. However, these values are close to the parameters for medium risk.

Regarding the Social Division of Labor scale, the scores show a predominance of medium and high psychosocial risk. The following variables were associated with high risk: being separated, divorced, or widowed; having completed postgraduate studies; being a nurse; working in the coronary ICU; working the night shift; having worked for more than 10 years at the institution; not having another employment relationship; working 41 to 50 hours per week; not taking breaks during the shift; not having experienced discrimination, prejudice, or abuse in the workplace; and rating free time as “excellent.”

Regarding Management Style, the Individualist Style was most common, especially among individuals who feel disrespected by their immediate superior or are undergoing psychological/psychiatric treatment. Although overall results fell within medium risk values, notable differences emerged in the predominance of management styles across specific groups. The Individualist Style was more prevalent among men, those aged 18 to 44 years, individuals without children, those self-identified as White or Black, college graduates, those earning 3 to 5 minimum monthly salaries, with 5 to 10 years of service at the institution, who take 3 to 4 breaks per shift, use regular medication, and use psychoactive substances. Conversely, the Collectivist Style was more common among women, separated, divorced, and single individuals working in the coronary or general ICU during the night shift, without another employment relationship, who do not take breaks during their shift, have a history of occupational accidents/illnesses, and are union members.

Regarding the Meaninglessness at Work factor, there was a strong predominance of low psychosocial risk. For the Mental Exhaustion factor, medium risk was prevalent. However, high risk was observed among homosexual individuals and those rating their free/leisure time and food intake as “very poor.” Low psychosocial risk was observed only in workers aged 55 to 64 years, those who did not report their sexual orientation, widowed individuals, those with up to 1 year of employment at the institution, working 21 to 30 hours per week, who exercise regularly, and rate their free/leisure time and sleep as “excellent” and food intake as “good.”

Regarding the Lack of Recognition, Psychological Harms, Social Harms, and Physical Harms factors, values ranged from low to medium psychosocial risk. In these factors, specific variables yielded significant results, such as in the ‘sexual orientation’ variable, where homosexual individuals presented a medium risk across the analyzed factors, while heterosexual individuals and those preferring not to report their sexual orientation presented a low risk. Only the Physical Harms factor presented a medium risk for heterosexual individuals.

Regarding color/race, the White population presented a medium risk across these factors. The Black population presented a medium risk for the Lack of Recognition and Physical Harms factors, while other factors indicated low risk. The Brown (mixed race) population presented a medium risk for Physical Harms only, with all other factors at low risk.

When examining professional categories (nurses/practical nurses), both groups showed a low risk for Lack of Recognition. However, nurses presented a medium risk for Psychological Harms and Social Harms, while practical nurses presented a low risk. Both categories presented a medium risk for the Physical Harms factor.

Our analysis identified other variables indicating medium risk for these factors, including: age between 25 and 34 years; no children; completed postgraduate studies; earning 3 to 5 minimum monthly salaries; working 51 to 60 hours per week; taking 3 to 4 breaks per shift; feeling disrespected by the immediate superior; history of discrimination, prejudice, or abuse in the workplace; psychological/psychiatric monitoring in the last 12 months; use of regular medication; and rating free/leisure time as “very poor,” “poor,” or “fair,” sleep quality as “very poor” or “poor,” and food intake as “very poor.”

## DISCUSSION

The nursing profession has historically been dominated by female workers. This population often faces significant mental health demands and a high rate of absenteeism, largely due to factors such as precarious employment, undervalued roles, and the burden of double or triple shifts combined with domestic and family care.^^12^,^17^-^19^^ In this study, most participants were women. Overall, we found no major differences between men and women regarding PFWs as assessed by the PROART. However, female participants reported a higher frequency of experiences with prejudice, discrimination, and abuse in the workplace, although both sexes faced similar psychosocial risks.

Regarding abuse in the workplace, studies indicate a high rate of exposure among nurses, encompassing workplace violence (e.g., moral harassment, physical abuse, sexual harassment) as well as ageism, gender-based violence, and other forms of discrimination directed at other groups.^^12^,^20^,^21^^ As a consequence of workplace abuse, Fernandes et al.^^12^^ highlight “stress and depression, which can worsen in daily life, negatively impacting job satisfaction and recognition” (p. 222). Our study supports these assumptions, revealing significant risks-particularly related to Mental Exhaustion, Lack of Recognition, and Psychological, Social, and Physical Harms-for workers with a history of prejudice, discrimination, or abuse in the workplace.

Furthermore, regarding gender, our findings indicate an association between having children and reduced psychosocial risk among participants. This was frequently associated with low risk across the factors evaluated and related to the Collectivist Management Style. These data align with the study by Campos et al.,^^19^^ who reported a lower prevalence of mental illness in both men and women with children, compared to those without children.

Another factor of analysis refers to issues of race and ethnicity, as they represent important social markers linked to inequality and vulnerability in the work environment. Sousa & Araújo^^22^^ state that “Gender and racial inequalities shape differences in work characteristics and exposure to occupational risks throughout the life trajectories of White or Black men and women. These differences, in turn, can produce or aggravate health problems.” (p. 2).

While existing literature indicates that Brown (mixed race) and Black populations face greater exposure to psychosocial risks, our study’s data from the Work-Related Harms scale suggest that the White population had greater risks, particularly in Psychological Harms and Social Harms, while Physical Harms were more prevalent among the Black population. Our results also indicate that the White population seeks mental health services more frequently. However, to establish a direct relationship between color/race and access to mental health services - and thus, a greater recognition of psychological and social demands - further studies specifically targeting the population in our study are necessary.

Another important factor to consider is sexual orientation. In our analysis, the homosexual population showed a low psychosocial risk only in the Meaninglessness at Work factor. This group showed a significant predominance of the Individualistic Management Style and relevant risks across all other analysis factors, especially Mental Exhaustion. The homosexual population also reported the highest incidence of past discrimination, prejudice, or abuse in the workplace, with 80% undergoing psychological or psychiatric monitoring.

Regarding these factors, Juliani & Scopinho^^23^^ highlight the numerous barriers faced by the LGBTQIA+ population in the workplace. Beyond challenges in entering and remaining in the workforce, issues such as stigmatization, prejudice, embarrassment, retaliation, and other forms of abuse can generate several psychosocial costs for these individuals, which can lead to lack of motivation, social isolation, depressive states, and, in severe cases, attempted suicide.

Age, income, and length of service also emerged as significant factors that align with existing literature. Our results showed that younger workers had a higher prevalence of psychosocial risks related to Lack of Recognition and Psychological, Social, and Physical Harms, while older workers reported these issues less frequently. Kirchhof et al.^^11^^ suggest that this difference may be linked to work experience and greater knowledge of the context, meaning that more experienced workers have more resources to navigate workplace challenges. Almeida et al.,^^24^^ when examining relationship conflicts between teams and working conditions, also noted differences in age and time since graduation, stating that older professionals exhibit less anxiety related to work demands.

Regarding income, Kirchhof et al.^^11^^ noted that low wages are a significant source of dissatisfaction in nursing, often leading workers to hold multiple jobs. While our data are consistent with the authors’ statements, we observed that individuals earning 3 to 5 minimum monthly salaries showed the most data suggestive of psychosocial risks, in contrast to the authors’ findings that the prevalence of mental health demands is mainly observed in workers with lower incomes. It is important to note that, in our study, all respondents in this income range reported having more than one employment relationship.

Shift-based work schedules, particularly night shifts, are a notable characteristic of nursing, especially in hospital settings. Night shifts are often linked to mental illness and emotional exhaustion, primarily due to sleep disturbances and reduced quality of life.^^11^,^13^^

Our results indicate that professionals on both shifts face a medium risk for Mental Exhaustion and Physical Harms. The night shift showed a predominance of the Individualistic Management Style and a high risk for the Social Division of Labor factor, suggesting a close relationship between psychosocial risks and work organization. Contrary to existing literature, night shift workers showed a low risk of Psychological and Social Harms, factors directly related to quality of life.

In this respect, our findings highlight the importance of quality-of-life factors, indicating that workers who reported poor sleep, insufficient free time, and inadequate food intake also experienced higher rates of psychosocial risks, particularly Mental Exhaustion, Lack of Recognition, and Psychological, Social, and Physical Harms.

Another important factor to consider is the relationship between workers and their teams and managers. Issues such as relationship difficulties within the team, poor communication, conflicts, ethical concerns, lack of recognition, and inadequate social support, feedback, and leadership are frequently cited as sources of emotional and mental health demands.^^13^,^20^,^24^^ Our study highlights these relationship issues as a predominant factor of medium risk for those feeling disrespected by their immediate superior. Furthermore, we observed the predominance of an Individualistic Management Style, common in hospital settings,^^25^^ alongside relationship conflicts and complaints about the lack of recognition within the team in open-ended questions. The general ICU was the sector that showed the most evidence of these relationship issues.

On a positive note, our research participants showed a striking predominance of low psychosocial risk regarding Meaninglessness at Work. As Pousa & Lucca^^20^^ state,

“Health professionals are among the groups that feel most impacted by their psychosocial work environment. Yet, their strong identification with their work serves as a motivating factor to overcome difficulties, enhancing well-being [...] the meaning, significance, and commitment to their work are intrinsic factors for these professionals.” (p. 5).

The prevalence of positive results suggests that nursing workers in the institution’s ICUs view this factor as beneficial for their mental health in the workplace.

The results obtained in this study, especially those that do not align with existing literature, require further investigation for a deeper understanding of the potential causes and variables influencing how workers perceive and navigate their workplace experiences. This study had the limitation of being conducted with a limited number of ICU professionals; therefore, further research is warranted for a more comprehensive analysis of the phenomena identified here.

## CONCLUSIONS

Our findings indicate that while the professional categories studied did not show significant differences among themselves, they collectively highlighted specific dimensions of the organizational and relational contexts where suffering is present. This includes factors contributing to physical and mental health issues, as well as elements that either harm or protect workers and their work. This research design offers a valuable perspective for understanding PFWs in an integrated manner that can support diagnostic and intervention efforts that prioritize strengthening the relationship between health and work. It is our hope that the findings of this study will inform local initiatives aimed at protecting the health of health care workers - an emerging and urgent demand to ensure and promote the well-being of those who provide care through their work.
